# Neural Circuits for Cognitive Appetite Control in Healthy and Obese Individuals: An fMRI Study

**DOI:** 10.1371/journal.pone.0116640

**Published:** 2015-02-06

**Authors:** Jetro J. Tuulari, Henry K. Karlsson, Jussi Hirvonen, Paulina Salminen, Pirjo Nuutila, Lauri Nummenmaa

**Affiliations:** 1 Turku PET Centre, University of Turku, Turku, Finland; 2 Department of Radiology, Turku University Hospital, Turku, Finland; 3 Department of Surgery, Turku University Hospital, Turku, Finland; 4 Department of Endocrinology, Turku University Hospital, Turku, Finland; 5 Department of Biomedical Engineering and Computational Science, School of Science, Aalto University, Aalto, Finland; 6 Brain Research Unit, O.V. Lounasmaa Laboratory, School of Science, Aalto University, Aalto, Finland; Beijing Normal University, Beijing 100875, CHINA

## Abstract

The mere sight of foods may activate the brain’s reward circuitry, and humans often experience difficulties in inhibiting urges to eat upon encountering visual food signals. Imbalance between the reward circuit and those supporting inhibitory control may underlie obesity, yet brain circuits supporting volitional control of appetite and their possible dysfunction that can lead to obesity remain poorly specified. Here we delineated the brain basis of volitional appetite control in healthy and obese individuals with functional magnetic resonance imaging (fMRI). Twenty-seven morbidly obese women (mean BMI = 41.4) and fourteen age-matched normal-weight women (mean BMI = 22.6) were scanned with 1.5 Tesla fMRI while viewing food pictures. They were instructed to inhibit their urge to eat the foods, view the stimuli passively or imagine eating the foods. Across all subjects, a frontal cortical control circuit was activated during appetite inhibition versus passive viewing of the foods. Inhibition minus imagined eating (appetite control) activated bilateral precunei and parietal cortices and frontal regions spanning anterior cingulate and superior medial frontal cortices. During appetite control, obese subjects had lower responses in the medial frontal, middle cingulate and dorsal caudate nuclei. Functional connectivity of the control circuit was increased in morbidly obese versus control subjects during appetite control, which might reflect impaired integrative and executive function in obesity.

## Introduction

In our modern society humans are surrounded by food advertisement and palatable visual food cues are readily available and on display in supermarkets and restaurants. Despite complex homeostatic mechanisms that govern eating and appetite [1], the mere visual cues of palatable foods may trigger a strong urge to eat notwithstanding current nutritional state, thus momentarily overriding homeostatic control and previous conscious decisions regarding eating behavior [2]. As visual and olfactory food cues are omnipresent in our everyday environment, they may play a key role in driving increased food consumption by triggering appetitive behavior. This may also contribute to the increasing prevalence of obesity in the industrialized world [3]. Consequently, understanding the physiological and psychological mechanisms underlying volitional restraint of energy intake is critical in fighting the ‘obesity epidemic’.

The sight of food cues engages the brain’s reward circuit and it shows greater activation encountering palatable versus bland foods [2], especially in obese individuals [4,5]. It has been suggested that, in addition to a hypersensitive reward circuit, an imbalance between the reward circuitry and frontal brain systems supporting inhibitory control may be a feature of obesity [2,4–6]. It is widely accepted that, the pre-supplemental motor area (preSMA) and anterior cingulate cortex form the key regions in the brain’s cognitive inhibition network [7–9], typically measured by Go/No-Go task [10]. However, these regions may also support more general cognitive processes involved in inhibitory processes, such as memory retrieval and working memory [11]. As the aforementioned frontal regions are anatomically connected to basal ganglia and thus to the striatal reward circuit [11] – known to be hyperactive in obese individuals [2] – it is possible that they could serve cognitive appetite control as well. In line with this, prior studies on cognitive appetite control while viewing food pictures have indeed implicated the frontal brain regions’ involvement when thinking of long-term costs and benefits of eating the presented foods [12] and fronto-striatal activations during cognitive reappraisal tasks [13].

The evidence of the influence of obesity on the functioning of the fronto cortical appetite control systems remains mixed, nevertheless. Some studies have reported no effects of Body Mass Index (BMI) on cortical inhibitory circuits [12], while some have observed smaller [14] and others larger [13] responses in this circuit in obese individuals. Although prior studies have revealed that obesity alters functional connectivity of the reward circuit [2], our understanding of how obesity could influence *functional connectivity* of the cognitive control networks and the reward circuit has remained elusive. It is indeed possible that the previously reported elevated reward circuit responses to palatable foods in obese individuals [4–6] could reflect altered inhibitory connections from the frontal cognitive control regions, rather than mere differences in regional hemodynamic responses.

Here, we used functional magnetic resonance imaging (fMRI) to study brain circuits supporting cognitive appetite control in normal-weight and morbidly obese individuals. Participants viewed pictures of foods, while their task was to either inhibit their urges to eat, imagine eating the foods or watch the foods passively. We hypothesized that volitional control of appetite would engage the fronto-cortical circuits, and that obese individuals would have lower frontal activations reflecting failure to volitionally inhibit their urges to eat. In line with this, we also predicted that functional connectivity of the control circuit would be lowered in obese individuals.

## Materials and Methods

The study was conducted in accordance with the Declaration of Helsinki and approved by the Ethical Committee of the Hospital District of South-Western Finland (SleevePET2 NCT01373892, http://www.clinicaltrials.gov). All participants signed an ethical committee-approved, informed consent form prior to scans.

### 2.1. Participants

Twenty-seven neurologically intact morbidly obese subjects (*M*_*BMI*_ = 41.4, *SD*_*BMI*_ = 3.9) were recruited for the study (Table 1). Fourteen neurologically intact and age-matched normal-weight volunteer subjects (*M*_*BMI*_ = 22.6, *SD*_*BMI*_ = 2.7) were recruited as a control group (Table 1). Eating disorders, severe mental disorders and substance abuse were exclusion criteria for all participants. In Finland ca. 85% of patients undergoing bariatric surgery are female. Consequently, the study was conducted with female participants who matched the characteristics of the national target population.

**Table 1:** The characteristics of study participants.

	Morbidly Obese *n = 27 females*	Mean	SD	Healthy Controls *n = 14 females*	Mean	SD	Independent sample t-test p-values
Age (years)		42.1	9.3		44.9	11.9	0.47
Height (cm)		165.2	6.3		165.5	6.4	0.89
Weight (kg)		113.5	14.4		61.8	6.9	< 0.001
BMI (kg/m^2^)		41.4	3.9		22.6	2.7	< 0.001
Subjective hunger rating before the MRI scan (1–9)		4.3	2.4		3.9	2.3	0.63

### 2.2. Experimental Design for fMRI

Participants were instructed to refrain from eating and to drink only water for 3–4 hours prior to scanning. Before the MRI scans, participants rated their feelings of hunger (using a scale ranging from 1 = I’m not feeling hungry at all to 9 = I’m starving) to assess the nutritional state [15].

**Fig 1:**
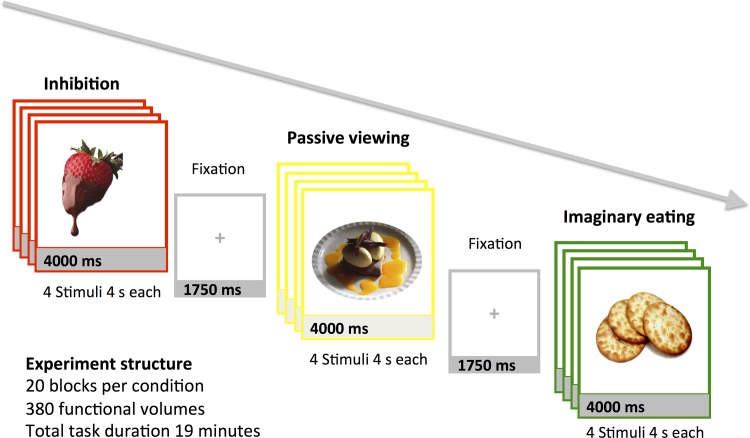
Experimental design and trial structure.

Stimuli and design are summarized in Fig. 1. The stimuli were 80 digitized full-color images of foods [2]. During fMRI acquisition, food stimuli were presented at the center of the screen in 16 s blocks intermixed with 1.75 s rest periods between blocks. During each block, participants saw four food pictures shown for 4 s each. A colored rectangle around the picture denoted the task the participant had to perform throughout each block. During *inhibition* condition, they had to inhibit urges to eat the food, during *passive viewing* condition they had to view the foods passively, and during *imaginary eating* condition they had to imagine eating the foods. The blocks were presented in a fixed, pseudo-randomized order, which was counterbalanced across participants. Altogether there were twenty blocks of each condition, and total scanning time was approximately 19 minutes. Participants were given written and spoken instructions before entering the scanner, and they practiced the task in the scanner before the experiment began. The participants were also interviewed after the experiment to assure that they had finished the task as instructed.

The stimulus presentation and behavioral data collection were controlled with Presentation software (Neurobehavioral Systems, Inc.). Stimuli were projected from an LCD projector onto a non-magnetic screen mounted at the foot of the bore, and an angled mirror reflected images onto the screen into the participants’ field of vision.

### 2.3. fMRI Acquisition and Analysis

MR imaging was performed with Philips Gyroscan Intera 1.5 T CV Nova Dual scanner at Turku PET centre. Whole-brain functional data were acquired with T2* weighted echo-planar imaging (EPI) sequence, sensitive to the blood-oxygen-level-dependent (BOLD) signal contrast (TR = 2987 ms, TE = 50 ms, 90° flip angle, 192 mm FOV, 64×64 reconstruction matrix, 62.5 kHz bandwidth, 4.0 mm slice thickness, with no gaps between slices, 30 interleaved slices acquired in ascending order). High-resolution anatomical images (1 mm^3^ resolution) were acquired using a T1-weighted sequence (TR 25 ms, TE 4.6 ms, flip angle 30°, 280 mm FOV, 256x256 reconstruction matrix).Altogether 380 functional volumes were acquired. Furthermore, four ‘dummy’ volumes were acquired and discarded at the beginning to allow for equilibration effects. The dummy volumes were not included in the analysis. Data were pre-processed and analyzed using SPM8 software (http://www.fil.ion.ucl.ac.uk/spm/) running on Matlab 2011b. The EPI images were realigned to the first scan image by rigid body transformations to correct for head movements. Echoplanar and structural images were co-registered and normalized to the T1 standard template in MNI space (Montreal Neurological Institute – International Consortium for Brain Mapping) using linear and non-linear transformations, and smoothed with a Gaussian kernel of 8 mm full width at half maximum—FWHM 8 mm.

### 2.4. Analysis of regional effects

A whole-brain random effects model was used. This two-stage process (first and second level) assesses effects on the basis of inter-subject variance and thus allows inferences about the population that the participants are drawn from. For each participant, we used a general linear model (GLM) to assess regional effects of task parameters on brain activation. The first level model included all three experimental conditions (inhibition, passive viewing and imaginary eating) as well as the six realignment parameters as effects of no interest. Low-frequency signal drift was removed using a high-pass filter (cut-off 128 s) and we applied autoregressive AR(1) modeling of temporal autocorrelations. The individual contrast images were generated using the t-contrasts i) inhibition minus viewing, ii) imaginary eating minus viewing, and iii) inhibition minus imaginary eating, as well as the opposite contrasts. The contrast images of the voxel-wise difference in beta estimates for the contrasts of interest. The second-level analysis used these contrast images in a new GLM from which generated statistical images, that is, SPM t-maps across all subjects and between subject groups. When the second-level analysis has balanced designs at first level with similar numbers of similar events for each subject it closely approximates a true mixed-effects design, exhibiting within- and between-subject variance. In addition, second-level analysis in SPM is considered to be robust against unequal group sizes, when unequal variances are assumed. The inhibition minus passive viewing and imaginary eating versus passive viewing contrasts were used to delineate the brain regions involved in cognitive control of appetite and mental processing of the hedonic value of the foods. The appetite control contrast (inhibition minus imaginary eating) was considered the main contrast of interest for revealing the neural basis of appetite control, as this enabled contrasting the two active food-related tasks directly against each other, thus accounting for effects of increasing task complexity in the active versus passive viewing conditions [11]. Data were thresholded at *p* < 0.05, false discovery rate (FDR) corrected at the cluster level.

### 2.5. Psychophysiological interactions (PPI) in the general linear model (GLM)

The connectivity between brain regions can vary as a function of the psychological context [16]. This is known as a Psychophysiological Interaction (PPI). PPIs do not require a specific anatomical model. Rather, they reveal context-dependent connectivity of the source region with any possible target region(s). This means that PPIs indicate task-dependent interactions between regional brain systems; thus the PPI reveals which regions have more or less similar activity pattern (‘connectivity’) with the source region as a function of a specific contrast. As is true for other connectivity methods such as dynamic causal modeling, PPIs do not indicate the direction of causal influences between source and target regions, nor whether the connectivity is mediated by mono- or poly-synaptic connections, nor changes in structural neuroplasticity from epoch to epoch [17].

Source regions for the brain’s inhibition network were selected from a previous meta-analysis on inhibitory processing in Go/No-Go tasks [10, 11] and caudate nucleus, given its key role in anticipatory food reward [2] (see Table 2 for MNI coordinates). A spherical 8-mm region of interest (ROI) was drawn at these locations in inhibition minus passive viewing contrast to further delineate connectivity changes in the cognitive control network. The time-series for each participant was computed by using the first eigenvariate from all voxel time series in the defined ROI, and de-convolved using the PPI-deconvolution parameter defaults in SPM8 [18]. The PPI term was then calculated as the element-by-element product of the ROI in “neuronal time-series” and a vector coding for the selected contrast (1 for inhibition and -1 for passive viewing). This product was then re-convolved by the canonical hemodynamic response function (hrf). First-level PPIs were run to generate SPM contrast images similar to the first level GLM model, and these contrast images were analyzed and thresholded in the second-level model as described above.

**Table 2:** Seed regions used in the PPI analysis.

Region	Hemisphere	X	Y	Z
Middle Cingulum	R	6	21	38
Precuneus	R	11	-72	58
preSMA	R	4	25	38
preSMA	L	-2	16	67
Insula	R	36	19	-7
Caudate nucleus	R	15	22	13
Middle (lateral) frontal cortex	L	-42	14	42
Orbitofrontal cortex	R	40	40	-2

## Results

### 3.1. Task-evoked BOLD responses across all subjects

**Fig 2:**
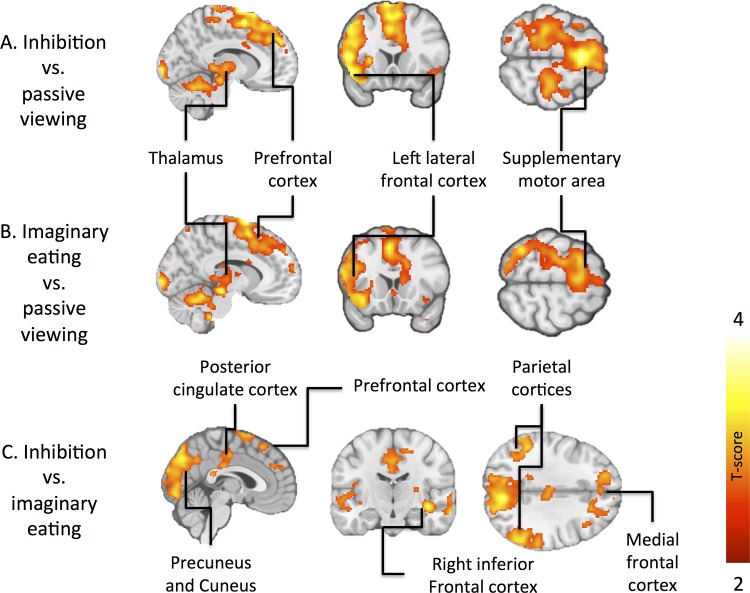
Regional brain activations across all subjects. Brain regions showing stronger responses during inhibition minus passive viewing condition (A), imaginary eating minus passive viewing condition (B) and inhibition condition minus imaginary eating (C). The data are thresholded at p < 0.05, FDR corrected at cluster level.

Contrasting the inhibition condition with passive viewing revealed widespread activation in fronto-cortical regions (Fig. 2A, Table 3). These included bilateral superior, superior medial- and middle frontal gyri, middle cingulate cortex and precentral gyrus bilaterally. Increased activation was also observed in the left inferior frontal gyrus and temporal pole. Supplemental motor area (SMA), thalamus and cerebellum were activated bilaterally. Additional activations were observed in the early visual areas in the occipital cortex. When imaginary eating was contrasted with passive viewing, similar brain areas were activated, such as in the inhibition minus passive viewing contrast (Fig. 2B, Table 3). Viewing versus inhibition and viewing versus imaginary eating conditions also both activated a similar pattern of brain regions: superior/middle frontal gyri, superior middle temporal gyri and precuneus (Table 4). Finally, we contrasted the inhibition and imaginary eating conditions directly with each other (appetite control). The areas showing increased activation during inhibition versus imaginary eating included right inferior frontal gyrus, bilateral middle frontal cortices, middle cingulate cortex, precuneus, cuneus, right hippocampus and parietal lobes (Fig. 2C, Table 3). The reverse comparison (imaginary eating > inhibition) showed increased activation in inferior temporal and superior parietal lobes (Table 3).

**Table 3:** Brain regions showing increased responses in all subjects during inhibition and imaginary eating versus passive viewing and inhibition versus imaginary eating.

Peak location	Hemisphere	X	Y	Z	T
Inhibition > Passive viewing					
Superior frontal gyrus	L	-18	52	32	6.59
Supplemental motor area	L	-2	6	60	6.40
		-8	2	74	6.28
Inhibition > Imaginary eating					
Inferior frontal gyrus (p. Triangularis)	R	50	24	6	5.54
Superior temporal gyrus	L	-46	-36	18	4.84
Superior temporal gyrus	R	64	-32	18	4.25
Cuneus	R	8	-74	32	4.50
Hippocampus	R	40	-16	-10	3.97
Angular gyrus	R	48	-62	40	3.93
Imaginary Eating> Passive viewing					
Cerebellum	R	27	-72	-24	5.39
Supplemental motor area	L	-12	4	72	6.02
Imaginary Eating > Inhibition					
Inferior temporal lobe	L	-50	-34	48	3.96
Superior parietal lobe	L	-24	-72	42	3.55

Data are thresholded at p < 0.05, FDR corrected at cluster level.

**Table 4:** Brain regions showing increased responses in all subjects during passive viewing versus inhibition and imaginary eating.

Peak location	Hemisphere	X	Y	Z	T
Passive viewing > Inhibition					
Middle cingulate cortex	R	6	-38	40	5.61
Middle temporal gyrus	R	44	-74	26	5.45
Middle frontal gyrus	R	40	8	46	4.08
		36	14	62	4.06
Superior frontal gyrus	R	30	20	62	4.05
Passive viewing > Imaginary Eating	Hemisphere	X	Y	Z	T
Superior temporal gyrus	R	56	-52	24	8.57
Middle temporal gyrus	R	52	-62	22	6.87
Precuneus	R	6	-76	38	6.47
Superior frontal gyrus	R	24	16	48	6.19
	R	38	52	24	5.25
	R	30	20	62	4.76
Superior temporal gyrus	L	-50	28	8	4.40
Middle temporal gyrus	L	-66	-28	8	3.72
	L	-60	-16	-2	3.69

Data are thresholded at p < 0.05, FDR corrected at cluster level.

### 3.2. Comparisons between normal-weight and morbidly obese subjects

**Fig 3:**
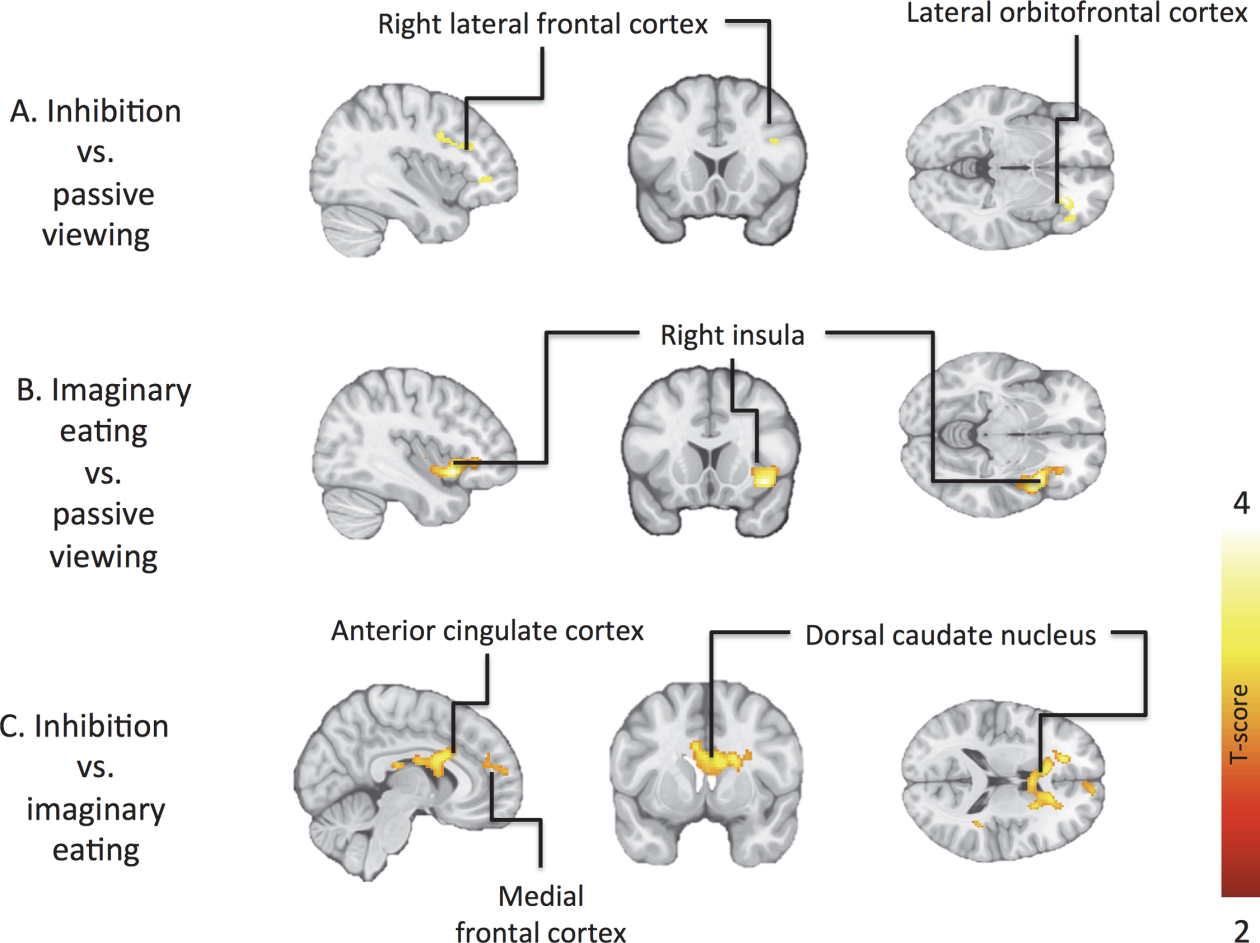
Regional differences in brain activations between normal-weight and obese subjects. Brain regions showing stronger activation in normal-weight versus morbidly obese subjects in inhibition minus passive viewing (A), imaginary eating minus passive viewing (B) and inhibition minus versus imaginary eating (C) contrasts. The data in C are thresholded at p < 0.05, FDR corrected at cluster level (p < 0.005, uncorrected at A and B).

In inhibition minus imaginary eating comparison, normal-weight subjects showed stronger activations than obese subjects in bilateral dorsal caudate nuclei and anterior cingulate cortex (Fig. 3C, Table 5). Correspondingly, obese subjects had greater activations in the bilateral posterior cingulum (Table 5). Other contrasts of interest revealed no significant activations at our a priori statistical threshold. Using a slightly more lenient statistical threshold (p < 0.005, uncorrected as compared to a priori threshold of p < 0.05, FDR corrected), we found that in inhibition vs. passive viewing comparison, normal-weight subjects had stronger responses in the left middle and inferior frontal gyri, and right orbitofrontal cortex (OFC), and obese subjects had greater activation of bilateral posterior cingulum (Fig. 3A, Table 5). When contrasting imaginary eating minus passive viewing, healthy subjects showed stronger right-hemispheric insular activation (Fig. 3B, Table 5).

**Table 5:** Brain regions showing significant between-group differences in the task-evoked BOLD responses.

Contrasts and comparisons	Hemisphere	X	Y	Z	T
Inhibition > Passive viewing *					
Healthy > Obese					
Middle orbital gyrus	R	28	32	-6	4.18
Inferior frontal gyrus (p. Orbitalis)	R	44	38	-2	3.26
Inferior frontal gyrus (p. Triangularis)	R	44	22	26	3.30
Obese > Healthy					
Posterior cingulate cortex	R	6	-38	12	5.16
	L	-4	-32	12	4.52
Imaginary Eating>Passive viewing*					
Healthy > Obese					
Insula	R	38	14	-10	3.74
Inhibition > Imaginary Eating					
Healthy > Obese					
Anterior cingulate cortex	L	-14	16	30	3.74
Caudate nucleus	R	20	6	22	2.99

Data are thresholded at p < 0.05, FDR-corrected at the cluster level. Note: * p<0.005 and cluster level uncorrected.

### 3.3. Psychophysiological interactions

**Fig 4:**
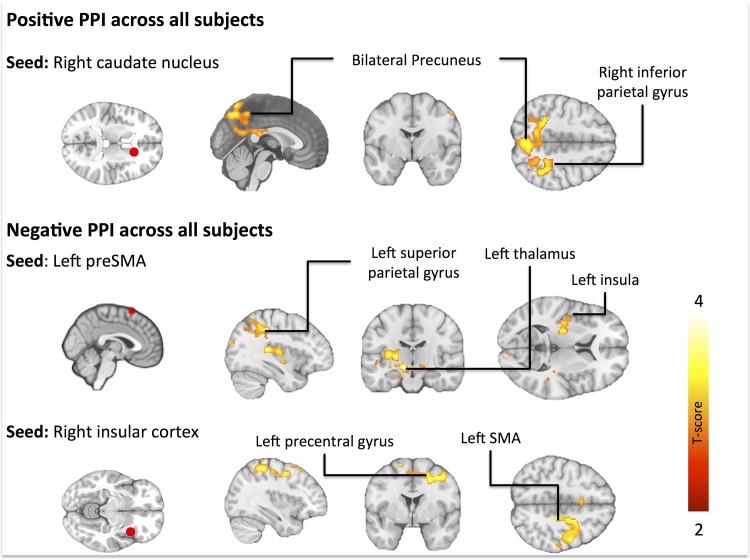
Functional connectivity (PPI) across all subjects. The data are thresholded at p<0.05, FDR corrected at cluster level.

Across all subjects, the right caudate nucleus showed increased task-driven (inhibition versus passive viewing) connectivity with bilateral precunei and cunei and parietal cortices (Fig. 4). Inferior parietal gyrus was the peak area of activation (Table 6). The left preSMA showed decreased connectivity across all subjects with bilateral cerebellum, left superior parietal gyrus (Table 6). The cluster extended to left insula, thalamus and caudate (Fig. 4). The right insula showed decreased connectivity across all subjects with the left pre-central gyrus and paracentral lobule (Table 6), with an extending cluster to preSMA (Fig. 4).

**Table 6:** Results of the functional connectivity analysis.

Region	Hemisphere	X	Y	Z	T
Across all Subjects (positive PPI)					
Seed: Right Caudate				
Inferior parietal gyrus	R	40	-48	48	3.82
Across all Subjects (negative PPI)					
Seed: Right Insula					
Paracentral lobule	L	-4	18	56	3.84
Precentral gyrus	L	-16	-16	72	3.73
Seed: Left Middle frontal gyrus					
Cerebellum	L	-12	-58	-14	3.97
Cuneus	R	2	-94	22	3.34
Superior parietal gyrus	L	-28	-64	54	3.06
Obese > Healthy					
Seed: Left Middle frontal gyrus					
Putamen	R	32	-20	4	4.09
Middle temporal gyrus	R	30	-64	6	3.38
Supplemental motors area (SMA)	R	8	-8	64	3.12
Seed: Right precuneus					
Supplemental motor area	R	10	-20	66	4.71
Pre-central gyrus	L	-20	-30	68	4.19
Inferior frontal Gyrus (p. Opercularis)	R	48	12	12	4.19
Seed: Right preSMA					
Precuneus	L	-8	-54	36	4.81
Middle cingulate cortex	R	10	16	36	4.15
Angular gyrus	R	42	-66	48	3.76
Inferior parietal lobule	R	52	-56	46	3.67

Data are thresholded at p < 0.05, FDR-corrected at the cluster level.

**Fig 5:**
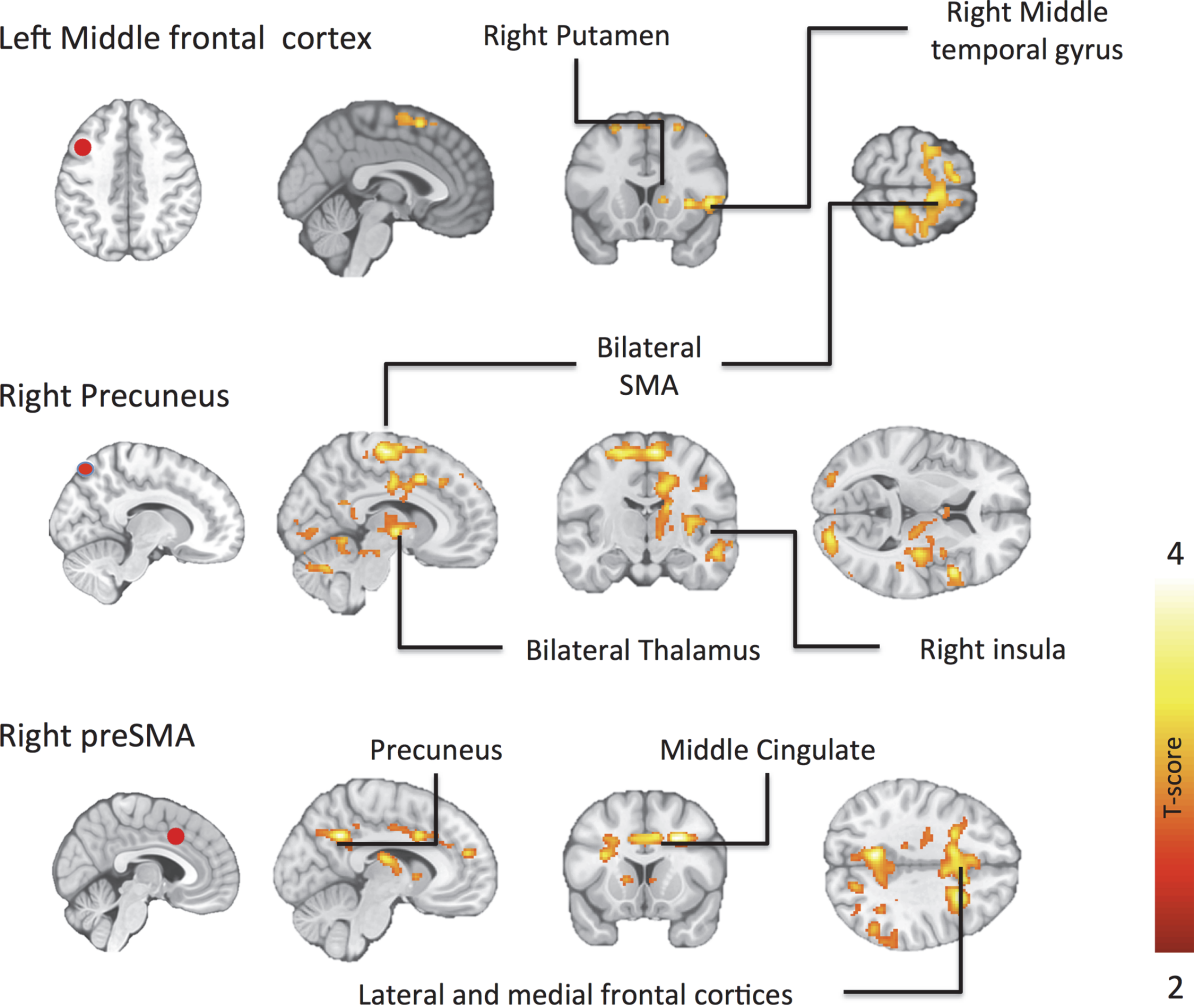
Differences in functional connectivity (PPI) in obese versus normal-weight individuals. The data are thresholded at p < 0.05, FDR corrected at cluster level.

Between-group comparisons revealed that obese subjects had stronger functional connectivity between middle frontal cortex and bilateral supplemental motor area (SMA), right putamen and right middle temporal gyrus (Fig. 5, Table 6). Obese versus normal-weight subjects also showed stronger connectivity between the right precuneus and bilateral supplemental motor area, bilateral thalamus, bilateral pre-central gyri and right inferior frontal gyrus (Table 6) extending to right insula (Fig. 5). In addition, obese versus normal-weight subjects showed stronger connectivity between preSMA and bilateral middle cingulate cortex, bilateral medial and lateral superior frontal cortices, thalamus, right inferior parietal lobule and right angular gyrus (Fig. 5, Table 6).

Hunger ratings (Table 1) did not differ between groups. Nevertheless, all second-level models were run separately with hunger rating as a regressor of no interest. This revealed that hunger did not significantly influence the results of the analyses reported in the manuscript.

## Discussion

Here we show that volitional appetite control while viewing visual food cues activates a network of prefrontal, frontal and parietal and inferior cerebellar cortical regions. This confirms that the fronto cortical control system – well known for inhibition and error monitoring [8,9] is also engaged during volitional appetite control. Moreover, regional responses and connectivity profiles of this network was influenced by obesity. During appetite control, obese individuals showed diminished responses in the frontal cortices as well as in the dorsal striatum, whereas during imaginary eating their responses were diminished in the insular cortex. Functional connectivity analysis further revealed dissociation between the regional responsiveness and interconnectivity of the cognitive control network in obese versus normal-weight individuals: connectivity of the control network, particularly of preSMA and precuneus, with regions involved in conflict monitoring (anterior cingulate cortex) and arousal control (thalamus) was stronger in obese versus normal-weight individuals.

### 4.1. Brain basis of cognitive appetite control

Eating and appetite are controlled by complex homeostatic mechanisms [1]. In real-life situations, a sudden urge to eat may be triggered by mere pictures of foods in advertisements, supermarkets and on TV; thus these impulses need to be inhibited to prevent excessive food intake. We found that frontal and prefrontal regions are reliably activated when individuals engage volitionally in such inhibition. These areas are likely the key components responsible for volitional appetite control, and are also implied in previous studies on response inhibition [8–11, 19–21]. However, a subset of these regions such as Broca’s area and left lateral frontal cortices were also engaged during imaginary eating. Both volitional inhibition of appetite and imaginary eating tasks require working memory in retrieving the task and some verbal instructions as well, so activation in these regions can be expected in both tasks [10]. Observed cerebellar activation could reflect engagement of a fronto-cerebellar network, which constantly updates anticipatory control mechanisms [22].

These findings from the task-evoked BOLD responses to cognitive appetite control accord with those made by Yokum et al. They compared the neural responses of three cognitive reappraisal strategies when adolescent subjects viewed visual food cues and thought of the long-term costs or benefits of not eating the food and suppressing cravings for the food. They found increased activation in inhibitory prefrontal and superior frontal regions during these mental tasks, yet in that study participants’ BMI did not modulate the intensity of brain activation [12]. Furthermore, Yokum et al reported decreased activation of the precuneus and posterior cingulate gyrus during task performance and more so during thinking of the long-term benefits for not eating the food as compared to other tasks [12]. In another recent study participants had to either increase or decrease appetite while viewing food pictures [13]. In the appetite decrease condition the subjects were asked to reappraise the presented stimuli i.e. give new meaning to the food picture such as picturing it to be a non-food item. Both appetite decrease and increase activated the striatum, insula and dorsolateral frontal regions as compared to watching across all subjects and more so in the obese subjects [13]. In the current study participants were not asked to adhere to a specific strategy during the appetite inhibition cognition. Consequently, the results reflect the general effects of appetite control, rather than those of a specific strategy (such as thinking about the consequences of eating). Taken together, it seems that the dorsolateral prefrontal cortex activation is consistently linked with volitional appetite control, whereas engagement of other components of the cognitive control and reward circuits may depend on the specific strategy used for controlling the appetite.When the two active cognitive task conditions (inhibition and imaginary eating) were contrasted with each other, we found that appetite control resulted in stronger activation the in medial frontal cortices, right inferior frontal gyrus, preSMA, posterior cingulum, cuneus / precuneus, bilateral middle frontal gyri, and occipital and superior parietal cortices (Fig. 2C, Table 3). The frontal brain areas correspond well with those implicated in response inhibition in prior work [8–11, 19–21]. The precuneus has a central role for a wide spectrum of highly integrated tasks, such as visuo-spatial imagery, episodic memory retrieval and self-processing operations [23]. In general, goal-directed action causes decreases in the tonic activity of precuneus during engagement to non-self-referential actions [23,24]. Because appetite control is goal-directed and likely involves accessing representations of one’s current homeostatic state, this kind of self-relevant processing during appetite control could explain the increased activation in the precuneus. Functional connectivity analysis revealed that the inhibition task increased connectivity between the caudate nucleus and parietal cortices, areas involved in attention control (Fig. 4). This likely reflects the increased attentional demands of the appetite control task, resulting in stronger coupling between the parietal cortices and the striatal regions encoding the hedonic value of the foods. On the contrary, appetite inhibition decreased connectivity between left preSMA-and-parietal cortex and right insula-and-left preSMA, respectively (Fig. 4). It could be speculated that engaging the control circuit diminishes the connectivity with associative and interoceptive areas correspondingly, thus reducing the effects of hedonic sensations and incentive motivation [25–27].

### 4.2. Obesity and cognitive control of appetite

During the inhibition versus imaginary eating condition, normal-weight individuals showed stronger responses than obese individuals in medial frontal and orbitofrontal cortices, as well as in dorsal caudate nucleus (Fig. 3). With more lenient thresholding, we also found that normal-weight individuals had stronger responses than obese individuals in the left dorsolateral prefrontal cortex and right orbitofrontal cortex during inhibition versus passive viewing condition and right insula during imaginary eating versus passive viewing condition (Fig. 3). These dampened responses in the cognitive control circuit in obesity are in line with prior imaging studies [13] and in general accord with the view that dysfunctional frontal brain systems supporting inhibitory control may underlie pathological eating in obesity [2, 4–6]. Decreased striatal and insular activation in obese individuals during appetite control, in turn, may reflect altered processing of incentive value of the presented food cues [25–27] due to chronically elevated substriatal stimulation following chronic excessive food intake.

Against our predictions, functional connectivity analysis revealed *enhanced* rather than diminished connectivity of the appetite control network in obese versus normal-weight individuals (Fig. 5). Obese individuals had stronger functional connectivity of pre-SMA with regions involved in conflict monitoring (anterior cingulate cortex) and arousal control (thalamus) during the appetite control task, suggesting that obese individuals’ control circuits may be hyperactive due to increased neural demands to inhibit excessive food intake. Furthermore, the obese individuals showed increased connectivity of precuneus with supplemental motor area, left postcentral gyrus and precentral gyrus. This could imply compensatory increase in control-related connectivity during volitional appetite control between right precuneus and prefrontal areas.

Altogether these findings imply that modulating the brain activity of the whole inhibitory circuitry may prove effective to combat our urges to eat excessively. Indeed, most of the currently available anti-obesity pharmaceuticals modulate widespread neurotransmitter systems [28]. Interestingly, an anti-obesity drug bupropion administered prior to viewing a food-cue video, enhances activation of brains regions implicated in cognitive appetite control in the current study (anterior cingulate, superior frontal areas, insula, superior parietal areas) [29]. This suggests that bupropion might exert, at least in part, its effects through supporting the inhibitory (superior frontal and prefrontal areas) and integrative (insula) brain circuitry upon encountering food cues in a real-life environment.

### 4.3. Limitations

We did not measure blood glucose or insulin levels, which are shown to influence fMRI responses to food pictures [30]. However, sensations of hunger were controlled for and found not to influence brain responses, possibly because all participants were scanned 3–4 h after they last ate. Even though our sample was sizeable, it is possible that more differences associated with the obese phenotype could be established in larger studies. Selection of morbidly obese subjects with greater pathological eating behavior (binge eating, impulsive personality or recent fast weight gain) could add to the results obtained in the current study. Because our study involved only females, it is possible that the results may not be generalizable to males. Prior studies have indeed shown gender differences in cortical processes associated with appetite control [31]. We did not control for the menstrual cycle phase in the current study [32]. From the patient group, 13 subjects and from the control group 8 subjects had no menstrual cycle either due to hysterectomy, progestin prevention or postmenopausal status. One patient in each group had combination contraceptives in use and for the rest the phase of the menstrual cycle was distributed evenly.

## Conclusions

We conclude that premotor areas, superior frontal cortices and the precuneus support cognitive control of appetite upon encountering visual food cues. Although these areas seem to function similarly in obese and normal-weight individuals, dorsal striatal responses during volitional appetite control are reduced in obese individuals. This suggests that even though frontal inhibitory function seems to be largely preserved in obesity, altered reward and homeostatic signaling together with heightened functional connectivity within the cognitive control circuit likely play an important role in the pathophysiology of obesity, and drive excessive eating and contribute to development and maintenance of obesity.
